# Surveillance of Tumour Development: The Relationship Between Tumour-Associated RNAs and Ribonucleases

**DOI:** 10.3389/fphar.2019.01019

**Published:** 2019-09-13

**Authors:** Nadezhda Mironova, Valentin Vlassov

**Affiliations:** Institute of Chemical Biology and Fundamental Medicine, Siberian Branch of Russian Academy of Sciences, Novosibirsk, Russia

**Keywords:** tumour-associated RNA, extracellular miRNAs, tumour development, ribonucleases, RNA degradation

## Abstract

Tumour progression is accompanied by rapid cell proliferation, loss of differentiation, the reprogramming of energy metabolism, loss of adhesion, escape of immune surveillance, induction of angiogenesis, and metastasis. Both coding and regulatory RNAs expressed by tumour cells and circulating in the blood are involved in all stages of tumour progression. Among the important tumour-associated RNAs are intracellular coding RNAs that determine the routes of metabolic pathways, cell cycle control, angiogenesis, adhesion, apoptosis and pathways responsible for transformation, and intracellular and extracellular non-coding RNAs involved in regulation of the expression of their proto-oncogenic and oncosuppressing mRNAs. Considering the diversity/variability of biological functions of RNAs, it becomes evident that extracellular RNAs represent important regulators of cell-to-cell communication and intracellular cascades that maintain cell proliferation and differentiation. In connection with the elucidation of such an important role for RNA, a surge in interest in RNA-degrading enzymes has increased. Natural ribonucleases (RNases) participate in various cellular processes including miRNA biogenesis, RNA decay and degradation that has determined their principal role in the sustention of RNA homeostasis in cells. Findings were obtained on the contribution of some endogenous ribonucleases in the maintenance of normal cell RNA homeostasis, which thus prevents cell transformation. These findings directed attention to exogenous ribonucleases as tools to compensate for the malfunction of endogenous ones. Recently a number of proteins with ribonuclease activity were discovered whose intracellular function remains unknown. Thus, the comprehensive investigation of physiological roles of RNases is still required. In this review we focused on the control mechanisms of cell transformation by endogenous ribonucleases, and the possibility of replacing malfunctioning enzymes with exogenous ones.

## Tumour-Associated RNAs and Their Role in Carcinogenesis

Tumour development is accompanied by rapid cell proliferation, loss of differentiation, the reprogramming of energy metabolism, loss of adhesion between tumour cells and matrix, evasion of immune surveillance, angiogenesis induction, infiltration growth, and metastatic spreading (Hanahan and Weinberg, 2011). Tumour-associated RNAs play an important role at all stages of tumour progression: intracellular coding RNAs determine the route of metabolic pathways (Zheng, 2012), cell cycle control, angiogenesis, adhesion, apoptosis (Suizu et al., 2000; Wadehra et al., 2005; Rocnik et al., 2006; Segura et al., 2007; Liu et al., 2013; Yan et al., 2016) and pathways responsible for transformation, such as PI3K/AKT (phosphatidylinositol-3-kinase/protein kinase B), TGF-β (tumor growth factor beta), JAK/STAT (Janus kinase/Signal transducer and activator of transcription) and MAPK (mitogen-activated protein kinase) (Ikushima and Miyazono, 2010; Ciuffreda et al., 2014; Thomas et al., 2015; Doi et al., 2017; Ouyang et al., 2017). Intracellular and extracellular non-coding RNAs participate in the regulation of the expression of target proto-oncogenic and oncosuppressive mRNAs as well (Dalmay and Edwards, 2006; Esquela-Kerscher and Slack, 2006).

Different groups unequivocally demonstrated the presence of various RNAs, including tumour-derived and tumour-associated RNAs, in the blood samples of patients with oncological diseases (Wieczorek et al., 1985; Kopreski et al., 1999; Vlassov et al., 2010). The range of RNAs discovered in blood plasma samples is rather wide and includes fragments of ribosomal RNA (rRNA), messenger RNA (mRNA), transport RNA (tRNA), mitochondrial RNA, small non-coding RNA particularly miRNA being detected in plasma or serum in RNA/protein complexes and within extracellular membrane vesicles (EV) (Valadi et al., 2007; Savelyeva et al., 2016; Savelyeva et al., 2017).

Data are accumulated that EV of cancer origin comprise pathogenic components, including mRNA and miRNA, that together with other components such as DNA proteins, transcriptional factors and lipids can take part in paracrine signalling in the tumour microenvironment (Fujita et al., 2016). These evidences also predict the role of EV-mediated transfer of cancer-associated biomolecules to distant organs contributing to the initiation of pre-metastatic niche formation. In support of this, an increasing number of publications has demonstrated the contribution of EV RNA in events of cancer development, namely, cell proliferation (Hong et al., 2009; Kogure et al., 2011; Zhang et al., 2015a), drug resistance (Challagundla et al., 2015), angiogenesis (Kosaka et al., 2013), immune modulation (Fabbri et al., 2012), and pre-metastatic niche formation (Fong et al., 2015).

### Extracellular Circulating mRNAS

Among the extracellular miRNA (ex-miRNA), mRNA fragments encoding tumour-associated antigens were detected, i.e. proteins that are expressed at low level in the cell, but overexpressed upon tumour progression. In the blood samples of patients with breast and thyroid cancer, malignant melanoma, and hepatocellular carcinoma, elevated levels of mRNA fragments encoding the telomerase components HTR (telomerase RNA component) and HTERT (telomerase reverse transcriptase) were detected (Chen et al., 2000; Novakovic et al., 2004). Overexpression of telomerase in cells can lead to epithelial-mesenchymal degeneration and tumour progression.

High levels of mRNA encoding mammaglobin, CK-19 (keratin 19) (Silva et al., 2001) and HER2/neu (Erb-B2 receptor tyrosine kinase 2) (Nicolini et al., 2017), which are specific markers of breast cancer (Lianidou et al., 2015), are also found in the blood plasma of patients with given disease. CK-19 is involved in maintaining the stability of epithelial cells and high expression levels are detected in tumour tissue (Lianidou et al., 2015). The HER2/neu oncogene is amplified at a high level in approximately 20% of all breast cancer cases and is related to rapid tumour proliferation and a poor prognosis.

In the case of lung cancer, the elevated levels of mRNA fragments encoding HER2/neu, hnRNP-B1 (heterogeneous nuclear ribonucleoproteins B1) and 5Т4 (oncofetal trophoblast glycoprotein) were detected in blood (Kopreski et al., 2001; Fleischhacker et al., 2001; Sueoka et al., 2005). hnRNP-B1 was found to play a significant role in the splicing of tumour suppressors and is considered an oncogene. It has also found to be crucial in the development of glioblastoma, hepatocellular carcinoma and lung cancer (Zech et al., 2006; Golan-Gerstl et al., 2011; Shilo et al., 2014). Oncogene 5T4 is involved in the modulation of cell adhesion and is over-expressed in many types of cancer cells, as along with such oncogenes as MYC (proto-oncogene c-Myc) and NRAS (NRAS proto-oncogene, GTPase).

For patients with malignant melanoma, the presence in blood of mRNA encoding for tyrosinase, MAGE-3 (melanoma-associated antigen 3), MCAM (melanoma cell adhesion molecule), p97 (transitional endoplasmic reticulum ATPase) and HMBS (hydroxymethylbilane synthase) is typical (Hoon et al., 2000; Hasselmann et al., 2001). Tyrosinase is involved in the synthesis of melanin, MAGE-3 is involved in malignant transformation and is the main tumour-specific melanoma antigen, and MCAM is involved in cell adhesion.

### Circulating Non-Coding RNAs

The pool of non-coding RNAs discovered in the bloodstream consists of long non-coding RNAs (lncRNA), short non-coding RNA including microRNAs (miRNA), and the recently discovered piwi-interacting RNAs. Long non-coding RNAs and piwi-interacting RNAs are described in detail in a recent review (Rapisuwon et al., 2016). Here we will focus on ex-miRNAs, which are important in the regulation of tumour development.

In 2008, the presence of extracellular miRNAs (ex-miRNA) in human blood was demonstrated by several research groups for the first time (Chim et al., 2008; Chen et al., 2008; Lawrie et al., 2008; Mitchell et al., 2008). In blood plasma, the main component of the ex-miRNAs are preserved enclosed in the ribonucleoprotein complexes comprising Ago2 protein, microvesicles and apoptotic bodies, and high and low-density lipoproteins (Valadi et al., 2007; Hunter et al., 2008; Zernecke et al., 2009; Vickers et al., 2011; Turchinovich et al., 2011; Arroyo et al., 2011; Bayraktar et al., 2017; Pucci et al., 2018). In this regard, miRNAs are highly stable and resistant to blood ribonucleases and can be transferred throughout the body, which makes their regulatory function virtually unlimited.

It is worth mentioning that miRNAs regulate fundamental cellular processes, such as proliferation, differentiation, metabolism, DNA repair, apoptosis, and transformation and can function as mediators in cell-to-cell communication, thereby acting like hormones (Ambros, 2004; Bartel, 2004; Croce and Calin, 2005; Calin and Croce, 2006; Cortez et al., 2011; Anfossi et al., 2018).

Circulating ex-miRNAs include both oncomirs and oncosuppressors. Attempts have been made to use elevated levels of particular miRNAs in the blood of patients with various tumours as biomarkers to diagnose the disease (Chen et al., 2008; Cortez et al., 2011; Cui et al., 2013; Lin et al., 2017; Pendlebury et al., 2017; Wang et al., 2017; Elghoroury et al., 2018). High levels of miR-21-5p were found in the blood of patients with colorectal cancer, gastric cancer, pancreatic ductal adenocarcinoma, and metastatic breast cancer (Wang et al., 2013; Muller et al., 2014; Khan et al., 2016; Emami et al., 2019). miR-155 is indicative of chronic lymphocytic leukaemia, breast cancer, and rectal cancer (Ferrajoli et al., 2013; Gao et al., 2017; Orosz et al., 2018). miR-125b-5p is found in blood of patients with metastatic breast cancer, non-small cell lung carcinoma and diffuse large B-cell lymphoma (Yuan et al., 2016; Cui et al., 2013).The miRNAs miR-125a-5p, miR-145, and mir-146a can be indicative of non-small cell lung cancer (Wang et al., 2015). Other miRNAs can also be indicative of other forms of cancer: miR-125a-3p – colon cancer (Wang et al., 2017), miR-200c and miR-141 – metastatic breast cancer (Zhang et al., 2017a); and the miR-200 family – prostate cancer and high-grade serous epithelial ovarian cancer (Lin et al., 2017; Pendlebury et al., 2017).

miR-21 represents one of the first microRNAs being defined as an oncomir, that regulate multiple tumour suppressors like the PTEN (phosphatase and tensin homolog), PDCD (programmed cell death), p53 (tumor suppressor p53) and TP63 (tumor protein p63) pathways (Meng et al., 2007; Asangani et al., 2008; Papagiannakopoulos et al., 2008). miR-155 act as an oncogene by inhibition of suppressor of cytokine signaling 1 (SOCS1) expression (Jiang et al., 2012). Taking into account the well-known functional link between inflammation and cancer, and the fact that inflammation to some extent is mediated by miR-155, the oncogenic role of miR-155 becomes clear (Jiang et al., 2010). However, although there is a lot of evidence for the oncogenic role of miRNA-155, it can also act as a tumour suppressor (Higgs and Slack, 2013).

Besides oncomirs, increased levels of which are identified in the blood of patients with various oncological diseases, tumour suppressor miRNAs can also be found in the blood stream. In this regard two or more miRNAs are used as diagnostic (by the level of oncomirs) and prognostic (by the level of tumour suppressor miRNAs) markers (Yang et al., 2015; Elghoroury et al., 2018).

The levels of tumour suppressor miRNAs are usually decreased in the blood of cancer patients, such as miRNAs belonging to let-7 family. Specifically, let-7 miRNAs directly interact with mRNAs encoding proteins involved in the cell cycle and signal transduction pathways that lead to carcinogenesis (Büssing et al., 2008). The decreased expression of let-7b is usually observed in lymph node metastases of breast cancer cells (Thammaiah and Jayaram, 2016). In addition, down-regulation of let-7b/g is evidenced during gastric cancer development being associated with poor survival and lymph node metastasis (Kang et al., 2014). A decreased level of miR-152 has been also detected in various human cancer cell lines and tumour tissues, such as gastrointestinal (Chen et al., 2010), endometrial (Tsuruta et al., 2011) and ovarian cancer (Zhou et al., 2012), as well as hepatocellular carcinoma (Huang et al., 2010), indicating that miR-152 might act as a tumour suppressor in these tumours.

### Role of Tumour-Associated Extracellular RNA (ExRNA) in Transformation

The secretion of RNAs in ribonucleoprotein complexes (RNP) by cells, and the transfer of those RNP between mammalian cells, was for the first time established in the 1970s (Kolodny et al., 1972). RNAs in RNPs can be a specific product released from tumour cells that may mediate host-tumour interaction and regulation of gene expression (Rosenberg-Nicolson and Nicolson, 1994). The recent discovery of regulatory RNAs, particularly miRNAs, has led to a revolutionary hypothesis that ex-miRNAs can mediate cell-to-cell signalling by paracrine or even endocrine manner, especially playing a crucial role in the context of cancer and metabolism. This hypothesis arose because several research groups found a large amount of miRNA in the bloodstream and was largely supported by numerous subsequent publications demonstrating that miRNAs in RNPs or EV can enter the recipient cells, change gene expression, and cause functional effects (Valadi et al., 2007; Skog et al., 2008; Mittelbrunn et al., 2011; Montecalvo et al., 2012).

Cell-to-cell communication by ex-miRNA has also been proven for cells of the immune system. Exosomal-transfered pro-inflammatory miR-155 and immunosuppressive miR-146a from dendritic cells was demonstrated to reduce the level of their mRNA targets, and reprogrammed the response of cells-recipients to endotoxin (Alexander et al., 2015). The mechanism of regulation of the activity and differentiation of mast cells was shown to be mediated by the transfer of mRNA and miRNA in exosomes between cells (Valadi et al., 2007). Recent studies have demonstrated that adipose-derived EV-circulating miRNAs participate in cell–cell crosstalk between adipose and liver tissues by altering mRNA expression and translation in target tissue (see review Thomou et al., 2017).

Communication between tumour and normal cells provides a route for tumours to manipulate their environment, making it more favourable for growth and invasion. Glioblastoma cells have been shown to secrete exosomes containing mRNA, miRNA, including oncogenic miR-21, and angiogenic proteins being uptaken by normal cells, including microvascular endothelial cells of the brain (Skog et al., 2008). One example is the destruction of tight contacts in the epithelial cells of blood vessels under the action of exosome-derived miR-105 secreted by neighbouring cancer cells, and the subsequent increase in metastasis (Zhou et al., 2014). EV-derived miRNA-21 secreted by hepatocellular carcinoma cells participates in tumour progression triggering the conversion of hepatocyte stellate cells to cancer-associated fibroblasts (Zhou et al., 2018). MiR-210 obtained from hepatocellular carcinoma cells as well, was found to promote endothelial cell migration along with angiogenesis, which was confirmed by the correlation between the elevated level of miR-210 in the blood of patients with hepatocellular carcinoma and high microvessel density (Lin et al., 2018). Thus, tumour-derived miRNAs are the tools for the transformation of the normal cells to malignant, and adjustment of their microenvironment for favorable tumor development.

## The Role of Endogenous Mammalian RNases in the Control of Intracellular Events and Signalling Pathways Responsible for Transformation

Benner hypothesized that a certain balance between RNAs, RNases, and ribonuclease inhibitors controls tissue development in higher organisms (Benner, 1988). Many factors participated in regulation of gene expression at the mRNA level. These factors are non-coding RNAs, RNA-binding proteins and RNases that maintain RNA-homeostasis in cells and discard aberrant RNAs through the degradation and turnover of transcripts, control of RNA decay, and biogenesis of miRNAs. Disruption of mentioned processes is mainly associated with distortion of expression or the improper functioning of these factors followed by cell transformation and tumour development. Among these factors, RNases play quite important role since they regulate the turnover of various transcripts at every stage of the cell cycle and participate in the processing of RNA involved in translation control.

**Figure 1:**
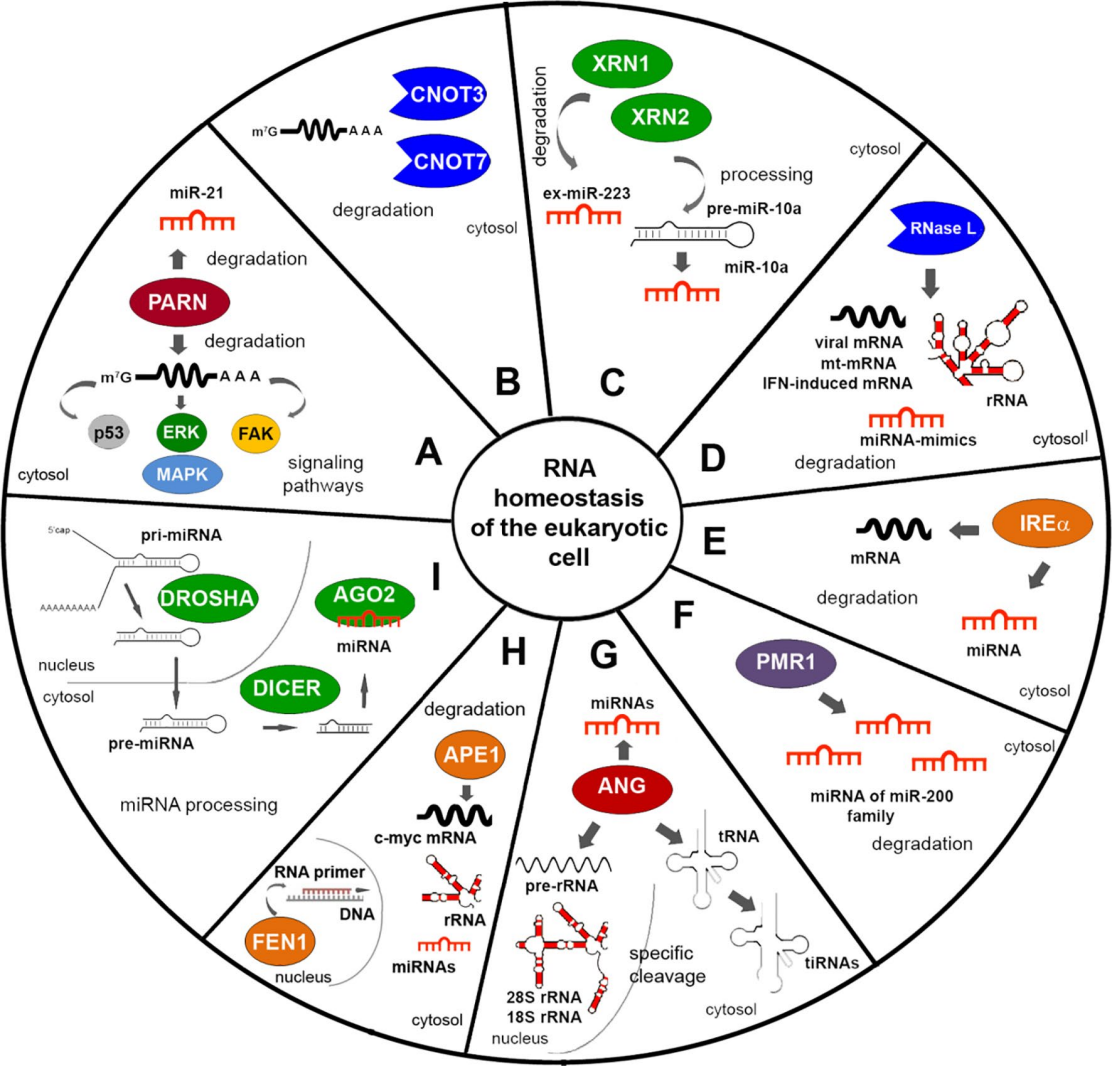
Endogenous RNases providing the maintenance of RNA homeostasis in the eukaryotic cell. **(A**–**C)** RNases of conventional RNA decay. **(D**–**F)** Stress signal induced RNases. **(G)** Angiogenin. **(H)** Proteins with ribonuclease activity participating in maintenance of DNA integrity. **(I)** RNases of miRNA biogenesis. The figure shows the targets of RNases and their activity at the level of RNA. Targets for GRBPs are presented in detail in rev. Alam and Kennedy (2019).

In a number of studies up to 2009, it was shown that intracellular RNases are involved in both induction and suppression of tumour progression (see review Kim and Lee, 2009). Nowadays a lot of information has appeared expanding the supervisory function of exogenous ribonucleases in the RNA world (**Tables 1** and **3**) and their intracellular RNA-targets (**Figure 1**).

**Table 1:** Endogenous RNases and other proteins with ribonuclease activity participating in maintenance of normal RNA homeostasis of eukaryotic cells.

RNases	Function	Intracellular role	Reference
CNOT3	deadenylase	Conventional RNA decay	Bartlam and Yamamoto, 2010
CNOT7	deadenylase		
PARN	poly(A)-specific RNase		Martinez et al., 2000
XRN1	5′–3′ exonuclease		Long and McNally, 2003
XRN2	5′–3′ exonuclease		Miki and Grosshans, 2013
RNase L	2′-5′-oligoadenylate-dependent endoribonuclease	Stress signal induced RNases	Li et al., 1998
IRE1α	a serine/threonine kinase, an endoribonuclease		Sidrauski and Walter, 1997
PMR1	endoribonuclease		Kim and Lee, 2009
ANG	endonuclease	RNA metabolism Neovascularization events	Sheng and Xu, 2016
G3BP1	RAS-GTPase-activating protein (SH3-domain)-binding protein	Regulation of mRNA stability and translation	Atlas et al., 2004 Taniuchi et al., 2011b; Winslow et al., 2013
APE1	apurinic/apyrimidinic endodeoxynuclease	DNA repair RNA turnover	Vascotto et al., 2009
FEN1	flap endonuclease	DNA replication RNA turnover	Shen et al., 2005
Drosha	endoribonuclease	RNases involved in miRNA biogenesis	Murchison and Hannon, 2004
Dicer	endoribonuclease		
Ago2	endoribonuclease		

CNOT3, CCR4-NOT transcription complex subunit 3; CNOT7, CCR4-NOT transcription complex subunit 7; PARN, poly(A)-specific ribonuclease; XRN1, 5’-3’ exoribonuclease 1; XRN2, 5’-3’ exoribonuclease 2; RNase L, ribonuclease L; IRE1a, serine/threonine-protein kinase/endoribonuclease IRE1; PMR1, ATPase secretory pathway Ca2+ transporting 1; ANG, angiogenin, ribonuclease, RNase A family, 5; G3BP1, GTPase activating protein (SH3 domain) binding protein 1; APE1, apurinic/apyrimidinic endodeoxyribonuclease 1; FEN1, flap structure-specific endonuclease 1; Drosha, double-stranded RNA-specific endoribonuclease, nuclear; Dicer, double-stranded RNA-specific endoribonuclease, cytoplasmic; Ago2, Argonaute RISC catalytic component 2.

**Table 2:** Exogenous RNases of different origin displaying antitumor activity.

RNases	Superfamily	Origin
Pancreatic RNase A	RNase A	Mammals/*Bos taurus*
Seminal BS-RNase		
Onconase/Ranpirnase		Amphibian/oocytes of *Rana pipiens*
Barnase	RNase T1	*Bacillus amyloliquefaciens*
RNase Sa 3		*Streptomyces aureofaciens*
Binase		*Bacillus pumilus*

**Table 3:** Endogenous RNases and their role in cancer development.

RNase	RNA targets^#^	References^#^	*In vitro*/*in vivo* effects*	References*
Endogenous RNases				
**Conventional RNA decay RNases**				
CNOT3	poly-A tails of mRNA	Bartlam and Yamamoto, 2010	*Tumour suppressor* T-cell acute lymphoblastic leukemia *Oncogene* non-small cell lung cancer drosophila eye cancer model	De Keersmaecker et al., 2013 Shirai et al., 2019 Vicente et al., 2018
CNOT7			*Oncogene* metastasis in mouse breast cancer model	Faraji et al., 2016
PARN	mRNAs involved in p53, FAK and MAPK signaling oncogenic miR-21	Lee et al., 2012; Devany et al., 2013	*Tumour suppressor*	Devany et al., 2013; Boele et al., 2014
XRN1	ex-miRNA degradation (ex-miRNA-223)	Zangari et al., 2017	*Tumour suppressor* osteogenic sarcoma	Zhang et al., 2002
XRN2	processing of pre-miR-10a to mature miR-10a	Zhang et al., 2017b	*Oncogene* lung cancer	Zhang et al., 2017b
**Stress signal induced RNases**				
RNase L	viral RNA, rRNA, mitochondrial mRNA, the interferone-induced mRNA exogeneous miRNA-mimics	Wreschner et al., 1981; Li et al., 1998; Le Roy et al., 2001; Nogimori et al., 2019	*Tumour suppressor* human prostate cancer PC3 cells prostate cancer	Banerjee et al., 2015; Dayal et al., 2017
IRE1α	mRNA and miRNA its own mRNA, mRNA *XBP1*, mRNA *CD59* and mRNAs involved in regulation of angiogenesis	Maurel et al., 2014 see rev. Kim and Lee, 2009	*Tumour suppressor* mutant forms of IREα in many types of cancer *Oncogene* transcriptional reprogramming in cancer cells gioblastoma	Parsons et al., 2008; Guichard et al., 2012; Chevet et al., 2016; Obacz et al., 2017; Logue et al., 2018
PMR1	miRNAs of the miR-200 family	Bracken et al., 2014; Gu et al., 2016; Perdigão-Henriques et al., 2016	*Oncogene* MCF-7 breast cancer cells mouse breast cancer cells	Gu et al., 2016; Perdigão-Henriques et al., 2016
**RNA metabolism, neovascularization**				
ANG	47S pre-rRNA 28S rRNA, 18S rRNA tRNA miRNA	Monti et al., 2009; Shapiro et al., 1986; Fu et al., 2009; Yamasaki et al., 2009; Sheng and Xu., 2016	*Oncogene* breast cancer prostate cancer colorectal cancer bladder cancer	Honda et al., 2015; Li et al., 2019; Etoh et al., 2000; Peres et al., 2016
**Proteins regulating mRNA stability**				
G3BP1	stabilize mRNA like mRNA *tau* and *CDK7* degradation of mRNA *MYC*, BART, *CTNNB1, PMP22, GAS5, IGF2*	Atlas et al., 2004; Gallouzi et al., 1998; Tourrière et al., 2001; Zekri et al., 2005; Taniuchi et al., 2011a; Taniuchi et al., 2011b; Winslow et al., 2013	*Oncogene* colon cancer thyroid cancer breast cancer head and neck cancer	Guitard et al., 2001; Barnes et al., 2002; French et al., 2002; Zhang et al., 2019
**Proteins with ribonuclease activity participating in maintenance of DNA integrity**				
APE1	*MYC* mRNA mRNAs (*in vitro*) rRNA miRNA	Barnes et al., 2009; Bergstrom et al., 2006; Vascotto et al., 2009; Antoniali et al., 2017b	*Tumour suppressor* c-myc overespressed tumors	Barnes et al., 2009
FEN1	RNA primers in (DNA replication)	Shen et al., 2005	*Oncogene* lung and prostate cancer	Sato et al., 2003; Lam et al., 2006
RNases involved in miRNA biogenesis**				
Drosha	pri-miRNAs	Murchison and Hannon, 2004	*Tumour suppressor* neuroblastoma endometrial cancer nasopharyngeal carcinoma gallbladder adenocarcinoma ovarian cancer melanoma blastemal Wilms tumour pineoblastoma *Oncogene* basal cell carcinoma squamous cell carcinoma smooth muscle neoplasms	Lin et al., 2010 Torres et al., 2011 Guo et al., 2012 Shu et al., 2012 Papachristou et al., 2012 Jafarnejad et al., 2013 Wegert et al., 2015 Snuderl et al., 2018 Sand et al., 2010
Dicer	pre-miRNA		*Tumour supressor* neuroblastoma endometrial cancer nasopharyngeal carcinoma gallbladder adenocarcinoma transitional cell carcinoma of the urinary bladder breast cancer lung cancer gastric cancer ovarian cancer	Lin et al., 2010 Torres et al., 2011 Guo et al., 2012 Shu et al., 2012 Wu et al., 2012 Khoshnaw et al., 2012 Karube et al., 2005 Zheng et al., 2007 Pampalakis et al., 2010
Ago2	mRNA		*Oncogene* breast invasive carcinoma colon and rectum adenocarcinoma bladder urothelial carcinoma prostate adenocarcinoma	Huang et al., 2014

^#^references are done in accordance with RNA target.

*references are done in accordance with in vitro/in vivo effects.

**data are presented in details in review (Hata and Kashima, 2016).

### RNases of Conventional RNA Decay

Deadenylation is an essential way of regulation of mRNA stability and expression of genes responsible for the fundamental functions such as development and differentiation at cell level under normal or pathological conditions, including chronic inflammation and cancer (Zhang et al., 2010; Zhang et al., 2015b). The CCR4-NOT complex, a major deadenylase in mammals, plays dual roles in the control of tumour development. The mammalian CCR4-NOT complex was described to comprise eight subunits: CNOT1, CNOT2, CNOT3, CNOT6 or 6L, CNOT7 or 8, CNOT9, CNOT10, and CNOT11 with four of them, namely, CNOT6/6L/7/8 and CNOT3 exhibited deadenylase properties (Bartlam and Yamamoto, 2010; **Figure 1B**).

Knockdown of CNOT3, a subunit incorporated in the CCR4-NOT complex and responsible for deadenylase activity, was shown to induce tumour development in a sensitized drosophila eye cancer model (Vicente et al., 2018). Moreover, mutations of the CNOT3 gene were discovered in samples of T-cell acute lymphoblastic leukemia (T-ALL) patients (De Keersmaecker et al., 2013), suggesting the tumour suppressor role of CNOT3. However, contrary to the tumour suppressive function components of the CCR4-NOT complex, it can have an impact on cancer progression. For instance, the up-regulation of CNOT3 promotes the progression of non-small cell lung cancer (Shirai et al., 2019) and activity of CNOT7 may stimulate migration of mouse breast cancer cells (Faraji et al., 2016; **Table 3**).

It is known that deadenylases facilitate miRNA-induced mRNA decay resulted from their interaction with the miRNA-induced silencing complex (miRISC). A vast amount of publications give an evidence of participation of deadenylation complexes, such as CCR4-NOT and Pan2–Pan3, in miRNA-mediated deadenylation being necessary for regulation of gene expression and stability of mRNA (see review Zhang et al., 2010). Poly(A)-specific ribonuclease (PARN) is an important deadenylase: among its targets are migration and adhesion factors, as well as mRNAs of proteins involved in p53, FAK (fokal adhesion kinase), and MAPK signaling (Lee et al., 2012; Devany et al., 2013; **Figure 1A**). PARN may also participate in miRNA-mediated deadenylation due to association with Ago2 in the RNA-induced silencing complex (RISC), and promote degradation of the oncogenic miR-21 followed by restoration of tumor suppressor activity of corresponding protein targets such as PTEN and p53 (Boele et al., 2014; Zhang et al., 2015a). PARN inhibition was shown to induce p53 accumulation and decrease cancer cell viability (Shukla et al., 2019).

5′–3′ exonuclease XRN1 is an enzyme being involved in conventional RNA decay (Long and McNally, 2003), which is also implicated in cancer as a tumour suppressor (**Table 3**). The decreased expression and/or complete depletion of XRN1 mRNA were found in primary samples of osteogenic sarcoma (Zhang et al., 2002). XRN1 realized additional control over epithelial to mesenchymal transition (EMT) on the level of ex-miRNA decay. It was found that XRN1 degrades ex-miRNA-223 derived from extracellular vesicles of polymorphonuclear leukocyte neutrophils after penetration into tumour cells, thus promoting transient epithelial-mesenchymal transition (Zangari et al., 2017; **Figure 1C**). Recently obtained data show that XRN1 negatively regulates autophagy in mammalian cells that thus reduces cell survival, which reinforces the evidence that this is a suppressor (Delorme-Axford et al., 2018). Contrary to this, 5′–3′ exonuclease XRN2 promotes EMT and metastasis through regulation of the processing of pre-miR-10a to mature miR-10a, and is a candidate inducer of spontaneous lung cancer (Zhang et al., 2017b; **Figure 1C**).

### Stress Signal Induced RNases

A number of endogenous RNases (RNase L, IRE1α, and PMR1) are normally silent in the cell and are induced under specific stress signals to effect tumour-modulating functions. Human RNase L displaying endoribonuclease activity expressed in many types of normal and cancerous mammalian cells (Zhou et al., 2005). RNase L is single-stranded ribonuclease able to cleave viral RNA, rRNA, and some cellular RNA both in cells and cell-free systems, at phosphodiester bonds in UU and UA sequences (Wreschner et al., 1981; Li et al., 1998; **Figure 1D**). In human and mouse cells, RNase L controls the stability of mRNA encoded in mitochondria and destabilizes the mRNA of genes induced by the interferon response to a viral infection (Li et al., 1998; Le Roy et al., 2001). RNase L is normally involved in innate immunity and antiviral defence (Malathi et al., 2007), however besides these functions it also plays a role as a tumour suppressor. Mutations in the RNase L gene were found to contribute to enhanced cell migration and invasion, and knockdown of RNase L in human prostate cancer cell line PC3 resulted in increase of tumour growth rate and metastases spreading *in vivo* (Banerjee et al., 2015; Dayal et al., 2017; **Table 3**). Cleavage of mRNAs encoding proteins involved in cell adhesion and migration appears a more likely mechanism for the inhibition of cell migration by RNase L (Banerjee et al., 2015). Interestingly, RNase L can discriminate and eliminate exogenous miRNA mimics (Nogimori et al., 2019; **Figure 1D**).

IRE1α is a serine/threonine kinase, an endoribonuclease, which is one of the major participants in endoplastic reticulum (ER) proteostasis and plays a dual role in cancer development (**Table 3**). It carries out both tumour-inducing and tumour-suppressing activity. Activation of IRE1α was observed in several types of tumors and was associated with overexpression of such oncogenes as BRAFV600E (mutant form V600E of B-Raf proto-oncogene, serine/threonine kinase gene), MYC, and HRAS (HRAS proto-oncogene, GTPase) (Croft et al., 2014). In turn, activation of IRE1α and its functioning as ribonuclease may lead to the process named RNA regulated IRE1-dependent decay (RIDD) that represent degradation of mRNA and miRNA targets (Maurel et al., 2014). In mammalian cells, the substrates for IRE1α are its own mRNA, mRNA encoding XBP1 and CD59, and other mRNAs encoding proteins involved in the regulation of angiogenesis (see review Kim and Lee, 2009; **Figure 1E**).

Several studies demonstrated that inhibition of the expression or the RNase activity of IRE1 suppresses the development of several types of tumours, mostly because of the ablation of pro-survival effects of XBP1 on tumour growth (Chevet et al., 2016; Obacz et al., 2017). Recently inhibition of IRE1 ribonuclease activity was found to influence the tumour cell secretome and enhance its sensitivity to chemotherapy (Logue et al., 2018). The tumour suppressive function of IRE1 was also detected. In several studies on genome screening, it was found that IRE1α is often found in the mutant form in various types of malignancies (Parsons et al., 2008; Guichard et al., 2012). Overexpression of IRE1 leads to a decrease in the expression of CD59, being implicated in the progression of lung cancer (Oikawa et al., 2007). Thus, IRE1 is an important RNase that exhibits a dual role in cancer progression by directing cancer progression and cell death.

PMR1 exhibits the properties of a proto-oncogene and is an effector of the EFGR (epidermal growth factor receptor) signalling pathway. Recently obtained data shows that increased migration activity and invasiveness of MCF-7 breast cancer cells is associated with high PMR1 activity, the targets of which are miRNAs of the miR-200 family, which are responsible for controlling adhesion and invasion (Bracken et al., 2014; Gu et al., 2016; Perdigão-Henriques et al., 2016; **Figure 1F**).

### Proteins Regulating mRNA Stability

RAS-GTPase-activating protein (SH3 domain)-binding proteins (G3BPs) represent a family of proteins capable of RNA binding and able to regulate mRNA stability and translation in response to environmental stresses (**Table 1**). The mammalian G3BP family consists of homologous proteins G3BP1, G3BP2a, and its splice variant G3BP2b with a similar molecular structure, which are located in the nucleus and cytoplasm. The different functions of G3BPs are summarized in a range of reviews (see revs Kim and Lee, 2009; Alam and Kennedy, 2019). From the point of view of its influence on the RNA world, it is important to note that GB3P1 participates in RNA metabolism including regulation of various cellular mRNAs and miRNAs. G3BP1 controls certain transcripts either due to its ability to stabilize mRNA like mRNA *tau* and *CDK7* (cyclin dependent kinase 7) (Atlas et al., 2004) or to cause mRNA degradation as in the case of mRNA MYC, BART (Epstein-Barr virus derived RNA encoding a set of miRNAs), *CTNNB1* (catenin beta 1), *PMP22* (peripheral myelin protein 22), *GAS5* (growth arrest specific 5), and *IGF2* (insulin like growth factor 2) (Gallouzi et al., 1998; Tourrière et al., 2001; Zekri et al., 2005; Taniuchi et al., 2011a; Taniuchi et al., 2011b; Winslow et al., 2013; **Table 3**). In earlier papers G3BPs were suggested to play a role in tumour development since their elevated levels were found in different types of proliferating cells and tumours (Guitard et al., 2001; Barnes et al., 2002; French et al., 2002). Moreover, G3BPs were found to be participants of key cell-growth associated molecular pathways important for tumorigenesis including RAS, the NF-κB, and MAPK pathways, and the ubiquitin proteasome system (Gallouzi et al., 1998; Prigent et al., 2000; Soncini et al., 2001; **Table 3**).

### Proteins With Ribonuclease Activity Participating in Maintenance of DNA Integrity

In addition, it was found that enzymes involved in DNA replication and repair, such as APE1, and FEN1, also exhibit RNase activity. Under stress, or when a nuclear localization signal is lost, these enzymes redistribute between the nucleus and the cytoplasm, where they can affect the level of cellular RNA. Two enzymes, APE1 and FEN1, have recently attracted close attention because of their ability to cleave RNA and the fact that their expression is associated with oncogenesis.

Apurinic/apyrimidinic endodeoxynuclease 1 (APE1) is an enzyme that exhibits both deoxyribonuclease and ribonuclease activity (**Table 1**). APE1 is mainly associated with DNA repair and redox regulation of transcription factors. In base excision repair (BER), APE1 functions as an apurinic/apyrimidinic endodeoxyribonuclease and corrects DNA damage caused by oxidizing or alkylating agents. APE1 was also found to exhibit endoribonuclease activity targeting *MYC* mRNA (Barnes et al., 2009; **Figure 1H**), and cleaving several other RNAs at UA, UG, and CA sites in the single stranded regions *in vitro* (Bergstrom et al., 2006). APE1 participates in rRNA quality control processes during cell division (Vascotto et al., 2009). Thus, APE1 performs several functions in the cell and can encourage genetic integrity and modulate turnover of different mRNAs as a ribonuclease. Recently, it has been suggested that this protein can perform non-canonical, but, nevertheless, important functions in RNA metabolism, regulating post-transcriptional expression of genes (Tell et al., 2010; Antoniali et al., 2014; Antoniali et al., 2017a).

Increased expression of APE1 was detected in a number of tumours: osteosarcoma (Wang et al., 2004), multiple myeloma (Yang et al., 2007), hepatocellular carcinoma (Di Maso et al., 2007), gastric cancer (Qing et al., 2015) (**Table 3**). APE1 is a normally a nuclear protein, but when cells acquire a cancerous phenotype it is redistributed between the nucleus and the cytoplasm (Jackson et al., 2005). There is evidence that the level of endoribonuclease activity of APE1 in the cytoplasm correlates with the aggressiveness of tumour. Of great interest are accumulating evidences demonstrating that APE1 may be involved in the control of gene expression due to its unsuspected activities during RNA metabolism (Antoniali et al., 2014; Jobert and Nilsen, 2014; Vohhodina et al., 2016) including miRNA expression (Antoniali et al., 2017b), thus enhancing APE1’s critical functions in tumour progression.

Human flap endonuclease 1 (FEN1), localized in the nucleus, exhibits endoribonuclease activity and is able to cleave *in vitro* both synthetic and natural RNA in double-stranded regions (Stevens, 1998). FEN1 functions include flap endonuclease activity resulting in the removal of RNA primers during DNA replication, 5’-3’-exonuclease activity, and gap-endonuclease and RNase H-like activities (Shen et al., 2005; **Figure 1H**). Similar to APE1, the FEN1 protein, in addition to its function of removing RNA primers during DNA replication, can also be involved in regulation of RNA level in a cell. In the development of tumours, FEN1 plays the role of an oncogene (**Table 3**). Overexpression of this protein is found in numerous aggressive fast-growing malignancies (Sato et al., 2003; Lam et al., 2006). There is a suggestion that the rate of RNA primer removal during DNA replication by FEN1 directly affects cell proliferation. So, in mouse models, it has been shown that FEN1 deficiency significantly contributes to the frequency and multiplicity of the occurrence of tumours (Kucherlapati et al., 2007).

### RNases Involved in miRNA Biogenesis

A wealth of data is accumulating that indicates a correlation between aberrant miRNA expression and tumorigenesis. Three RNases: Drosha, Dicer, and Ago are involved in miRNA biogenesis (Murchison and Hannon, 2004; **Figure 1I**), and, accordingly, disorders in their expression can influence cancer development. The increased levels of Drosha and Dicer, their intracellular redistribution, and malfunction, is observed in many types of cancer cells. Increased Drosha and Dicer levels also correlate with elevated levels of oncogenic miRNAs.

The biogenesis of miRNAs starts by RNA polymerase II (Pol II)-mediated transcription of the miRNA gene encoded in the genome. This process generates long primary (pri-miRNA) transcripts comprising a stem-loop hairpin structure (Kim et al., 2009). Drosha is an essential part of the microprocessor complex (with its cofactor DGCR8) that continues miRNA biogenesis *via* cleavage of pri-miRNAs with the formation of precursor miRNA (pre-miRNAs) (Kim et al., 2009; **Figure 1I**). Mutations in the Drosha/DGCR8 microprocessor complex subunit miRNA microprocessor complex are associated with high-risk of development of blastemal type Wilms tumours (Wegert et al., 2015). Reduced expression level of Drosha was found in melanoma (Jafarnejad et al., 2013), ovarian cancer (Papachristou et al., 2012), neuroblastoma (Lin et al., 2010), endometrial cancer (Torres et al., 2011), nasopharyngeal carcinoma (Guo et al., 2012), and gallbladder adenocarcinoma (Shu et al., 2012; **Table 3**). Recurrent homozygous deletions of Drosha were found in pineoblastoma (Snuderl et al., 2018). Single nucleotide polymorphisms (SNPs) in the sequence of Drosha gene were also found to correlate with high risk of cancer development (Wen et al., 2018). However, elevated levels of Drosha were found for a number of neoplasias: basal cell carcinoma, squamous cell carcinoma, and smooth muscle neoplasms (Sand et al., 2010).

The second processing step in miRNA biogenesis is realized by the cleavage of pre-miRNA with the RNase III Dicer endonuclease and RISC-loading complex subunit TRBP, which generates an approximately 22-nt miRNA duplex (Kim et al., 2009; **Figure 1I**). Dicer, an important RNase III endonuclease involved in miRNA processing, is down-regulated in many tumours, such as neuroblastoma (Lin et al., 2010), endometrial cancer (Torres et al., 2011), nasopharyngeal carcinoma (Guo et al., 2012), gallbladder adenocarcinoma (Shu et al., 2012), transitional cell carcinoma of the urinary bladder (Wu et al., 2012), breast cancer (Khoshnaw et al., 2012), lung cancer (Karube et al., 2005), gastric cancer (Zheng et al., 2007), colorectal cancer (Sun et al., 2017), and ovarian cancer (Pampalakis et al., 2010; **Table 3**). Low expression of Dicer is linked to poor prognosis and recurrence of cervical cancer (He et al., 2014). In addition, some correlations were found between the single nucleotide polymorphisms of Dicer and development and prognosis of several froms of epithelial cancers and endometrial cancer (Guo et al., 2012; Oz et al., 2018). Deletion of Dicer1 in a mouse model enhanced tumorigenesis (Kumar et al., 2009). The phosphorylation status of Dicer correlates with endometrioid tumour invasion (Aryal et al., 2019). Downregulation of Dicer expression was observed in human cancers and has been identified in promoting cancer metastasis and tumorigenesis due to repression of global miRNA maturation (Kumar et al., 2007; Martello et al., 2010).

Analysis of data from The Cancer Genome Atlas evaluated a significant influence of alterations in miRNA machinery genes on the development of multiple forms of malignancies. In particular, incidence of Ago2 alterations is the highest among the other miRNA-machinery genes and its contribution varies from 12.3% in case of colon and rectum adenocarcinoma to 20.7–23.30% in case of breast invasive carcinoma, bladder urothelial carcinoma, and prostate adenocarcinoma (Huang et al., 2014). The explanation may be due to the decreased competition between different miRNA species in regulation of gene expression and facilitation of operation of oncogenic miRNAs following the overexpression of Ago2 (Vickers et al., 2007).

## Exogenous Ribonucleases With Antitumor Activity and Mechanisms of Their Action

The antitumor potential of exogenous RNases has been studied for more than 60 years, due to their main function—the degradation of nucleic acids. Up to date, the most well-studied RNases with established antitumor activity are: BS-RNase from bull testes (Pouckova et al., 2004), amphibian RNase onconase from oocytes of Rana pipiens (Lee et al., 2000), bovine pancreatic RNase A (Patutina et al., 2010; Patutina et al., 2011), modified variants of RNase 1 from humans (Rutkoski et al., 2013) that belong to the RNase A superfamily, microbial RNase barnase from *Bacillus amyloliquefaciens* (Prior et al., 1996) and binase from *Bacillus pumilis* (Mironova et al., 2013a; Makeeva et al., 2017) relative to RNase T1 superfamily (**Table 2**).

A lot of published data confirm that exogenous RNases target different RNAs in a tumour cell. As already discussed, the degradation of RNAs managed by endogenous RNases plays a significant role in controlling gene expression, maturation, and turnover of RNA, which may be associated with malignant cell transformation and tumour progression. It can be assumed that exogenous RNases may restore the expression and/or activity of endogenous RNases disturbed in the tumour cell and modulate functions of tumour-associated RNAs. The mechanism of the cytotoxic action of exogenous RNases, presumed and partially confirmed in various studies, consists of series of stages. Firstly, the exogenous RNase binds with a tumour cell, is then internalised, gains access to the cytosol, and finally degrades intracellular RNA. The binding mechanisms of RNases with tumour cells, and their following penetration, are described in detail in a number of reviews (Makarov and Ilinskaya, 2003; Chao and Raines, 2011; Mit’kevich et al., 2014b); here we will focus directly on RNA degradation by exogenous RNases. It should be noted that in addition to intracellular RNAs, RNases, when released into the bloodstream, can also cause the degradation of circulating exRNA (Simsekyilmaz et al., 2014; Zernecke and Preissner, 2016; Lu et al., 2018).

It is obvious that the central molecular targets of RNases are various RNAs: rRNA, mRNA, RNA in the RNP complexes, tRNA, and non-coding long and small RNA. *In vitro* RNases destroy rRNA and tRNA in equal amounts, however, a certain type of RNA is more preferable *in vivo* for each RNase: BS RNase destroys rRNA (Mastronicola et al., 1995; Liao et al., 1996), while onconase preferentially degrades tRNA (Iordanov et al., 2000; Saxena et al., 2002; **Table 4**). Other intracellular targets of onconase are also rRNA, mRNA, and miRNA (Goparaju et al., 2011; **Table 4**). Thus, the toxic effect of RNases on tumour cells is associated with their main function - the ability to cleave RNA. However some data indicate that the ribonuclease activity of RNases is not the only component that provides an impact on their antitumor activity, but it is realized through the destabilization of double-stranded RNA (Sorrentino et al., 2003) or its irreversible binding (Blaszczyk et al., 2004).

**Table 4:** Exogenous RNases displaying antitumor activity.

RNase	RNA targets^#^	References^#^	*In vitro/in vivo* effects*	References*
*RNase A superfamily*				
bovine pancreatic RNase A	miRNA mRNA	Mironova et al., 2013b; Mironova et al., 2017	solid and ascitic tumors growth in mice and rats Lewis lung carcinoma Hepatoma A1	Ledoux, 1955a, Ledoux, 1955b Patutina et al., 2010; Patutina et al., 2011
bovine seminal BS-RNase	rRNA	Mastronicola et al., 1995; Liao et al., 1996	thyroid carcinoma SVT2 and 3T3 fibroblast cells ML-2 myeloid cells; neuroblastoma cells	Cinatl et al., 1999 Marinov and Soucek, 2000 Kotchetkov et al., 2001
Onconase/Ranpirnase	tRNAs pre-miRNA	Iordanov et al., 2000; Qiao et al., 2012	B cell lymphoproliferative disorders chronic myeloid leukemia lung carcinoma and pancreatic adenocarcinoma multiple myeloma, adenocarcinoma, and prostate cancer mesothelioma xenograft models non-small cell lung cancer and mesothelioma xenograft models	Mikulski et al., 1990a Ita et al., 2008 Turcotte et al., 2009 Nasu et al., 2011 Smolewski et al., 2014 Shen et al., 2016
*RNase chimeras*				
bovine pancreatic RNase A coupled to human transferrin or antibodies to the transferrin receptor	total cellular RNA, mainly rRNA, mRNA	Rybak et al., 1991; Mastronicola et al., 1995; Liao et al., 1996	K562 (human erythroleukemia-derived cell line)	Rybak et al., 1991
bovine pancreatic RNase A conjugated with mAb to the transferrin receptor or to the T cell antigen, CD5			glioblastoma xenograft	Newton et al., 1992
human pancreatic RNase 1 fused with human epidermal growth factor			squamous carcinoma cell line mouse melanoma cell line B16	Psarras et al., 1998 Futami et al., 1999
conjugates of BS-RNase with poly [N- (2-hydroxypropyl) methacrylamide]			xenografts of melanoma	Soucek et al., 2002
BS-RNase PHPMA conjugates			xenografts of melanoma, neuroblastoma and ovarian cancer	Pouckova et al., 2004
conjugate of transferrin with mutated variant of human pancreatic RNase hRNase (Gly89→Cys) and mutant eosinophil-derived neurotoxin	n.d.		U251 cell line (human glioma) Wehi 7.1 cell line(mouse T lymphoma)	Suzuki et al., 1999
immunoRNases on the base of variants of pancreatic human RNase fused with antibodies against ErbB2	n.d.		gastric tumor cells breast cancer cells resistant to trastuzumab	D’Avino et al., 2014
Conjugate of onconase with murine anti-CD22 antibody RFB4	tRNAs pre-miRNA	Iordanov et al., 2000 Qiao et al., 2012	non-Hodgkin’s lymphoma (pre-clinical model)	Rybak, 2008 Newton et al., 2009
Conjugate of onconase with chlorotoxin			mouse glioma xenograft model	Wang and Guo, 2015
*RNase dimers*				
bovine pancreatic RNase A dimer (mutant similar to BS-RNase)	n.d.		malignant, SV40 transformed SVT2 fibroblasts	Di Donato et al., 1994
human pancreatic RNase 1 dimer	total cellular RNA		human thyroid tumour- derived cells	Piccoli et al., 1999
*RNase T1 superfamily*				
Barnase	n.d.		carcinoma cell lines and human leukemia	Edelweiss et al., 2008
RNase Sa 3	n.d.		human erythroleukemia cells K-562	Sevcik et al., 2002
RNase Sa, mutants with enhanced positive charge	n.d.		acute myeloid leukemia Kasumi-1 cells	Mitkevich et al., 2014a
Binase	mRNA of oncogenes AML1-ETO, KIT	Ilinskaya et al., 2007; Mitkevich et al., 2011	human myelogenous leukemia cells R562, transgenic myeloid progenitor cells expressing activated KIT-oncogene human A549 alveolar adenocarcinoma cells mouse Lewis lung carcinoma, lymphosarcoma RLS_40_, melanoma B16 leukemic Kasumi-1 cells human ovarian cancer cells SiHa cervical carcinoma cells	Ilinskaya et al., 2007 Cabrera-Fuentes et al., 2013 Mironova et al., 2013a Sen’kova et al., 2014 Mitkevich et al., 2014a Garipov et al., 2014 Mitkevich et al., 2017
Conjugate of two barnase molecules with scFv of a humanized 4D5 antibody			human breast cancer xenografts	Balandin et al., 2011
Immunotoxins on the base of barnase, fused with MYC epitope, Pseudomonas toxin, Shiga-like toxin E.coli and Fc domain of human antibody IgGγ1			MYC-specific B-cells	Stepanov et al., 2011
Binase immobilized on halloysite nanotubes	mRNA of oncogenes AML1-ETO, KIT	Ilinskaya et al., 2007 Mitkevich et al., 2011	human colon adenocarcinoma cells.	Khodzhaeva et al., 2017

^#^references are done in accordance with RNA target.

*references are done in accordance with in vitro/in vivo effects.

n.d. – not detected.

PHPMA, Poly[N-(2-hydroxypropyl)methacrylamide]; ErbB2, Erb-B2 receptor tyrosine kinase 2; KIT, KIT proto-oncogene, receptor tyrosine kinase; AML1-ETO, fusion protein detectable in patients with acute myelogenous leukemia; RLS_40_, drug-resistant lymphosarcoma RLS_40_.

After penetration into the cell, RNases degrade cellular RNA, as a result of which protein synthesis is blocked and apoptosis is initiated. It was shown for binase that treatment of the cells by the enzyme results in a significant decrease in the total amount of RNA in the cells, which, nevertheless, does not correlate with the level of cytotoxic effect of binase (Mitkevich et al., 2010a). It has been suggested that changes in intracellular tumour-associated RNA levels observed after treatment with exogenous RNases may be the result of both direct degradation of mRNA and miRNAs that suppress the expression of certain genes, and/or generation of new siRNA-like molecules that can participate in the regulation of intracellular processes by the mechanism of RNA interference (Zhao et al., 2008; Saxena et al., 2009). Thus, the catalytic activity of exogenous RNases is considered a key factor in determining the regulation of intracellular processes involving RNA.

## Ribonucleases of RNase A Superfamily

### Bovine pancreatic RNase A and Pancreatic RNase 1 of Human

Bovine pancreatic ribonuclease A (RNase A) represents a small protein consisted of 124 amino acids with molecular weight equal to 13.7 kDa that, nevertheless, has the highest catalytic activity among the proteins of its superfamily. RNases belonging to RNase A superfamily catalyse the cleavage of RNA at phosphodiester bonds after pyrimidine residues in single-stranded regions (Raines, 1998). RNase A is the first ribonuclease whose antitumor activity was studied *in vitro* (Ledoux and Revell, 1955; Ledoux, 1956) and *in vivo* (Ledoux, 1955a; Ledoux, 1955b; Aleksandrowicz, 1958a; Aleksandrowicz, 1958b; Telford et al., 1959).

However, the obtained results were contradictory. This enzyme, at the doses of 40–1,000 mg/kg, caused retardation in the growth of solid and ascitic tumours in mice and rats (Ledoux, 1955a; Ledoux, 1955b). In the other studies, it was shown that RNase A does not exhibit cytotoxic and antitumor effects even using high doses of the enzyme injected into solid tumours (De Lamirande, 1961; Raines, 1998; Leland and Raines, 2001). Attempts were made to investigate the pancreatic RNase 1 of humans that belongs to the RNase A superfamily, and displays high catalytic activity, as an antitumor drug but the enzyme showed a very weak cytotoxic effect in cell cultures. The absence of cytotoxic activity of RNase A and RNase 1 was explained by their inactivation with intracellular ribonuclease inhibitor (RI), which form an extremely strong complex with these RNases (Kd < 10^-15^ ?) (Johnson et al., 2007).

In a number of studies, an increase in the cytotoxicity of RNase A and RNase 1 was achieved by conjugating these RNases with peptides, proteins and antibodies, which increased the efficiency of their capture by tumour cells (Rybak et al., 1991; Newton et al., 1992; Psarras et al., 1998; Futami et al., 1999). To obtain RI-resistant RNase A and RNase 1 variants, methods of protein engineering, chemical modification, or protein conversion with the formation of covalent dimers were used (Di Donato et al., 1994; Rutkoski et al., 2005; Rutkoski et al., 2011). By means of site-directed mutagenesis D’Alessio and colleagues developed artificial dimers of RNase A and RNase 1 that showed cytotoxicity towards cancer cells (Di Donato et al., 1994; Piccoli et al., 1999). This allowed the production of derivatives of RNase A and RNase 1 with high antitumor activity. High cytotoxic activity against cancer cells was achieved by the conjugation of transferrin with mutated variant of human pancreatic RNase hRNase (Gly89→Cys) and mutant eosinophil-derived neurotoxin (Suzuki et al., 1999). Recently obtained data revealed that RI-resistant variants of pancreatic RNase 1 of human displayed strong toxic effect toward lung cancer and melanoma cells and worked sinergically with protein kinases in the ERK pathway (Hoang et al., 2018). A number of immunoRNases on the base of variants of pancreatic human RNase were developed which being fused with antibodies against ErbB2 exhibit strong toxic effects to ErbB2-positive tumor cells (D’Avino et al., 2014).

Despite encouraging results, interest in the therapeutic potential of RNase A disappeared for a long time, but arose again several decades later, when RNase was able to exert cytotoxic effects on tumour cells at much lower doses than was used in the 1950s. In 2002, conjugation of RNase A with poly [N- (2-hydroxypropyl) methacrylamide] led to constructions that effectively suppressed the growth of melanoma in nude mice (Soucek et al., 2002). In 2004, the first information appeared on the cytotoxic effect of RNase variants, which were inactivated by RI (Naddeo et al., 2005). A cytotoxic variant of human pancreatic RNase PE5 containing a nuclear localization signal was developed, which, despite its sensitivity to RI, demonstrated high cytotoxicity on a panel of various tumour cells (Bosch et al., 2004). Further, additional modifications of the PE5 structure led to the appearance of variants of RNase 1 with high cytotoxicity (Vert et al., 2012).

From 2010 to the present, several clinical studies of pancreatic RNase A have been conducted to treat various types of tumours. The first study (Phase I, ClinicalTrials.gov Identifier: NCT01201018), conducted from September 2010 to June 2012, used RNase A in peroral form (O’Shadi R) for the treatment of patients with various cancers. Although the official report on this study has not yet been presented, four more clinical trials of RNase A have since started: for the treatment of metastatic non-small cell lung cancer (ClinicalTrials.gov Identifier: NCT02134990), mesothelioma (ClinicalTrials.gov Identifier: NCT01627795), basal cell carcinoma (ClinicalTrials.gov Identifier: NCT02007317), acute myeloid leukemia and lymphoid leukemia (ClinicalTrials.gov Identifier: NCT02462265). Variant of human pancreatic RNase 1 with 95% sequence identity named QBI-139 is studied in a phase I clinical trial for the treatment of advanced refractory solid tumors (ClinicalTrials.gov Identifier: NCT00818831).

Colleagues in our laboratory used several mouse tumour models to demonstrate that RNase A administered in very low doses exhibited antitumor and antimetastatic activity (Patutina et al., 2010; Patutina et al., 2011). Attempts to find molecular targets of RNase A in the tumour and blood of tumour-bearing mice (with the example of Lewis lung carcinoma) revealed that antitumor and antimetastatic action of RNase A is realized *via* degradation of extracellular circulating miRNAs and is accompanied by significant boost of miRNA synthesis in tumour tissue (Mironova et al., 2013b). The microRNA boost in the tumour was associated with the overexpression of genes involved in microRNA biogenesis such as *Drosha*, *Xpo5*, *Dicer*, and *Ago2*. Ribonuclease activity of RNase A was demonstrated to play crucial role both in antitumour/antimetastatic activity and the influence on the expression of microRNA and the microRNA processing genes.

Moreover, it was found that RNase A affected the whole transcriptome of murine Lewis lung carcinoma (Mironova et al., 2017) providing the downregulation of 644 transcripts and upregulation of 322 transcripts. Major part of the genes are involved in signalling pathways that maintain energy metabolism, promote cell growth and transformation, modulate the cancer microenvironment and extracellular matrix components as well as stimulate cellular proliferation and differentiation. As a result of RNase A treatment, we also detected an upregulation in carbohydrate metabolism, the stimulation of inositol phosphate cascade and oxidative phosphorylation as well as re-arrangement of apoptosis, transcription, cell cycle control and adhesion processes. Taken together, these data suggest that reorganization of the intracellular network of tumour cells caused by RNase A led to enhancement of energy cascade activity, shift in cancer-related cell growth and dissemination processes, and partial depletion of signalling pathways that have tumour-promoting activity (Mironova et al., 2017).

### Angiogenin

Angiogenin (ANG) was described as the first proto-oncogenes among ribonucleases, and for this reason, researchers have never tried to use it as an antitumor drug. Nevertheless, since ANG is one of the brightest representatives of the RNase A superfamily, although it belongs to endogenous RNase, we discuss it in this section. ANG participated in neovascularization events, and an increased level of its expression was noted in many types of cancer cells (see rev. Kim and Lee, 2009).

Originally, human ANG was isolated as an angiogenesis factor of tumour origin; however, the expression of ANG by cells of various tissues suggests that its functions are not limited to neovascularization. Similar to RNaseA, ANG cleaves RNA in single-stranded regions, displaying Pyr-X cleavage specificity (Russo et al., 1996), although the ribonuclease activity of ANG is 10^−5^−10^−6^-times lower than RNase A activity (Lee and Vallee, 1989). In spite of the very weak ribonuclease activity of ANG, this activity is important for its biological functions, allowing ANG to drive an orchestra of various RNAs in a cell (Yamasaki et al., 2009; Ivanov et al., 2011; Li et al., 2019).

However, ANG exerts its functions not only due to its own ribonuclease activity, but also due to ability to bind with certain promoter regions of DNA and histone proteins, thus acting as a chromatin remodelling activator. ANG induces the synthesis of rRNAs by binding with promoter region of ribosomal DNA (rDNA), thereby promoting transcription of the precursor of 47S rRNA (Sheng et al., 2014). ANG in the nucleus may be involved in regulation of mRNA transcription. Using a chromatin immunoprecipitation-chip assay, Sheng and colleagues identified 699 genes that may be regulated by ANG on the mRNA level, many of which are related to tumorigenesis, such as proteins of Wnt and TGF-β pathways (Sheng and Xu, 2016).

A number of data highlighted the contribution of cellular ANG to the metabolism of RNAs, both in the nucleus and cytoplasm. In experiments *in vitro* it was shown that the ability of nuclear ANG to cleave 28S and 18S rRNAs, which, together with data on its participation in cleavage of the first cleavage site (A0) of the 47S pre-rRNA, provides evidence that ANG may enhance rRNA processing (Shapiro et al., 1986; Monti et al., 2009; **Table 3**). In addition, ANG carry out an important function in the tRNA metabolism that takes place in the cytoplasm. Under conditions of oxidative stress, hypoxia, and starvation, ANG performs the cleavage of the conserved single-stranded 3′-CCA termini of tRNA or its anticodon loop inducing the formation of so named tiRNA (tRNA-derived, stress induced small RNA) (Yamasaki et al., 2009; **Figure 1G**). ANG-produced tiRNA plays a significant role in proliferation of breast and prostate cancer cells (Honda et al., 2015; **Table 3**). It was revealed that ANG enhances colorectal cancer growth and metastasis both in *in vitro* and *in vivo* systems, producing a higher level of a 5’-tiRNA from mature tRNA-Val (Li et al., 2019). The resulting tiRNAs reprogram the translation of proteins, promoting damage repair and cell survival (Ivanov et al., 2011). Among two tiRNAs generated by ANG, 5′-tiRNA (but not 3′-tiRNA) inhibits translation *in vitro*, however, not all 5′-tiRNA are active. Preliminary data from Sheng and colleagues shows that ANG can also participate in degradation of miRNAs (Sheng and Xu, 2016).

### Onconase/Ranpirnase (From Oocytes of Frog *Rana Pipiens*)

In the late 1980s, the Alfacell corporation conducted a study on an extract of oocytes or early embryos of the Northern Leopard Frog (*Rana pipiens*), which has profound cytostatic and cytotoxic activity towards tumour cells (**Table 2**). The active component of the extract was a protein of small size (11.82 kDa), being found in unfertilized oocytes as well. The amino acid sequence of this protein, originally named P-30, and later onconase or ranpirnase, resembled the sequence of enzymes of the RNase A superfamily (Ardelt et al., 1991). Onconase is the smallest protein of the RNase A superfamily having only 104 amino acids. Onconase displays significant ribonuclease activity that is 10^2^-10^5^ times lower in comparison with the activity of RNase A (Ardelt et al., 1991; Ardelt et al., 1994; Boix et al., 1996). Onconase exhibits the antiproliferative and cytotoxic activity through interference with cell-cycle regulation and induction of programmed cell death by a mechanism described in details previously (Lee and Raines, 2008; Porta et al., 2008).

Onconase is an extremely stable protein that is not inactivated by RI (Boix et al., 1996). In the beginning, the cytostatic and cytotoxic facilities of onconase were investigated on the cell lines of human HL-60 leukemia, carcinoma A-253, and Colo 320 CM colon adenocarcinoma (Darzynkiewicz et al., 1988). Onconase caused a retardation of cell proliferation by increasing the duration of the G1 phase of the cell cycle, accompanied by a reduction in the DNA replication frequency. Cytotoxicity of onconase was shown in tumour cell lines of different histogenesis: B cell lymphoproliferative disorders (Smolewski et al., 2014), chronic myeloid leukemia (Turcotte et al., 2009), lung carcinoma and pancreatic adenocarcinoma (Mikulski et al., 1990a), multiple myeloma, adenocarcinoma, and prostate cancer (Ita et al., 2008; **Table 4**).

Initial *in vivo* studies of onconase were performed on the Madison M109 carcinoma model of mice and it was demonstrated that the survival rate of tumour-bearing animals after treatment with onconase increases 12-fold compared with the control (Mikulski et al., 1990b). Recent studies on mesothelioma xenograft models have shown significant suppression of tumour growth by onconase (Nasu et al., 2011); studies on non-small cell lung cancer and mesothelioma xenograft models have shown suppression of tumour growth and angiogenesis when the combined action of onconase and dihydroartemisinin was used (Shen et al., 2016). A number of publications have demonstrated the antitumor activity of conjugates of onconase with antibodies specifically addressed the enzyme to tumour cells (Rybak, 2008; Newton et al., 2009; **Table 4**). In recent studies, a high antitumor activity of onconase conjugated with chlorotoxin has been shown on a mouse glioma xenograft model (Wang and Guo, 2015). The increase of cytotoxicity of onconase to tumour cells was reached *via* obtaining dimers of the enzyme (Fagagnini et al., 2017).

Onconase was one of the first ribonucleases studied in pre-clinical and clinical trials (Costanzi et al., 2005). Onconase has been approved for clinical use as an orphan drug for treatment of unresectable malignant mesothelioma in the United States, Europe, and Australia (Mikulski et al., 2002; Altomare et al., 2010). Clinical trials have shown that onconase is well tolerated by patients, has low immunogenicity, but has high nephrotoxicity (Mikulski et al., 2002). However, recent clinical trials of onconase for the treatment of patients with non-small cell lung cancer (ClinicalTrials.gov Identifier: NCT01184287) and mesothelioma (ClinicalTrials.gov Identifier: NCT00003034) have been prematurely terminated.

Although the main targets for onconase are tRNAs (Iordanov et al., 2000), an ability to affect miRNAs has also been found (Qiao et al., 2012; **Table 4**). The model of onconase-mediated cytotoxicity predicates that onconase is internalized in cytosol of tumour cells and cleaves tRNAs followed by ubiquitous inhibition of protein translation and apoptosis induction (Lee and Raines, 2008). Biochemical studies performed by Qiao and colleagues evaluate miRNAs as the direct downstream RNA targets of onconase. Onconase was found to downregulate miRNAs by cleavage of its precursor forms, thus decreasing the amount of mature miRNAs arisen from Dicer activity (Qiao et al., 2012). In addition, onconase was demonstrated to exert miRNA-mediated effects through downregulation of NF-kβ using specific miRNAs, particularly, upregulating miR-17 and downregulating miR-30c (Goparaju et al., 2011).

### BS-RNase (Bovine Seminal)

BS-RNase was revealed independently by Hosokawa and Irie in 1971 (Hosokawa and Irie, 1971), D’Alessio with colleagues in 1972 (D′Alessio et al., 1972), and Dostal and Matousek in 1972 (Dostal and Matousek, 1972). It is singular among all ribonucleases in that it has a quaternary structure. BS-RNase is a natural dimer that comprises a couple of identical subunits connected by two disulfide bonds and non-covalent interactions (D′Alessio et al., 1991). The amino acid sequence of the BS-RNase subunit and its structure classify this enzyme as belonging to the pancreatic RNase A superfamily (Beintema et al., 1988).

The polypeptide chain of the BS-RNase subunit contains 124 amino acids and has 80% homology with RNase A. Two cysteine residues located at 31 and 32 positions of the BS-RNase represents the most important difference between BS-RNase and RNase A. These two cysteines are involved in the formation of an intermolecular disulfide bond between Cys31 of one subunit and Cys32 of the second subunit, followed by dimerization of enzyme (Di Donato and D′Alessio, 1973). The dimeric enzyme (27.218 kDa) represents is a composition of two different quaternary forms, denoted as M = M and M × M (Piccoli et al., 1992).

The enzyme displays cytotoxic activity towards tumour cells only in dimeric form, and the ribonuclease activity is absolutely crucial (Kim et al., 1995a). However, the groups of D’Alessio and Raines showed that a single subunit of BS-RNase, which has a higher catalytic activity than the dimer, does not exhibit a cytotoxic effect on tumour cells (Vestia et al., 1980; Kim et al., 1995b). The explanation was that a separate subunit, but not a dimeric form of the enzyme, is inactivated by cytosolic RI (Murthy and Sirdeshmukh, 1992). In addition, the dimeric form of BS-RNase, but not monomeric, was shown to destabilize the membranes of tumour cells, and this destabilization contributes to the observed antitumor effect of the enzyme (Mancheño et al., 1994).

The antitumor activity of BS-RNase has been studied mainly on tumour cell lines and, to a lesser extent, on *in vivo* tumour models. BS-RNase exhibited a cytotoxic effect on various tumour cell lines: SVT2 and 3T3 fibroblast cells, ML-2 myeloid cells, neuroblastoma cells and thyroid carcinomas (Cinatl et al., 1999; Marinov and Soucek, 2000; Kotchetkov et al., 2001; **Table 4**).

Soucek and colleagues developed conjugates of BS-RNase with poly [N- (2-hydroxypropyl) methacrylamide], which protects the enzyme from degradation in the bloodstream, and demonstrated a significant inhibition of melanoma growth on nude mice, whereas intact BS-RNase was ineffective (Soucek et al., 2002). BS-RNase PHPMA conjugates also showed high efficiency in various human tumour models in CD-1 nude mice: melanoma, neuroblastoma, and ovarian cancer (Pouckova et al., 2004; **Table 4**).

### RNases of RNase T1 Superfamily (Bacterial and Fungal)

Recently, many RNases of bacterial and fungal origin have been discovered that exhibit cytotoxic activity (**Table 2**). In contrast to mammalian cytotoxic RNases, which belong to the RNase A superfamily, microbial RNases are related to the RNase T1 superfamily. The RNase T1 superfamily consists of 25 enzymes of fungal and bacterial origin that have a similar amino acid sequence and tertiary structure (Yoshida, 2001). RNases of the RNase T1 superfamily catalyse RNA cleavage at phosphodiester bonds after guanine residues (G↓X) in single-stranded regions.

Fungal RNases, being also denoted as ribotoxins (α-sarcin, mitogillin and restrictocin), perform the cleavage of the eukaryotic 28S rRNA of the large ribosome subunit at a single phosphodiester bond leading to the inactivation of protein synthesis, induction of apoptosis and cell death (Endo et al., 1983; Kao et al., 1998; Olmo et al., 2001; Lacadena et al., 2007). Although α-sarcin demonstrated high cytotoxic activity against a number of tumours, including sarcoma, it also displayed high hepatotoxicity and caused toxic heart damage in healthy animals.

The most well-known of the microbial ribonucleases that exhibit cytotoxic activity on tumour cells are RNase Sa (RNase from *Streptomyces aureofaciens*), barnase (RNase from *Bacillus amyloliquefaciens*), and binase (RNase from *Bacillus intermedius*; **Tables 2** and **4**). According to a recent genotypic identification, the strain known as *B. intermedius* belongs to the *B. pumilus* species, so it has been renamed accordingly (GenBank Accession No. HQ650161.1). Binase and barnase have no homology with mammalian RNases and are not recognized by RI (Rutkoski and Raines, 2008). Barnase and binase are small proteins that consist of 110 (12.382 kDa) and 109 (12.213 kDa) amino acids, respectively, with 85% structure homology (Hartley and Barker, 1972; Aphanasenko et al., 1979).

Cytotoxic effects of RNase Sa were demonstrated towards acute myeloid leukemia Kasumi-1 cells (Mitkevich et al., 2014a; **Table 4**). The Deyev group is engaged in a comprehensive study of the properties of barnase. The ability of barnase to eliminate malignant cells was shown in carcinoma cell lines and human leukemia (Edelweiss et al., 2008). For targeted delivery of enzyme into tumour cells, conjugates were obtained (Balandin et al., 2011; **Table 4**). These conjugates were consisted of two barnase molecules conjugated to a single-stranded variable fragment (scFv) of a humanized 4D5 antibody targeted to the extracellular domain of human epidermal growth factor receptor 2 HER2, which is overexpressed in many human carcinomas. On the basis of barstar, which is an inhibitor of barnase of bacterial origin, and barnase conjugated with fragments of various antibodies and nanoparticles, the development of multifunctional supramolecular structures for the elimination of malignant cells was proposed (Nikitin et al., 2010). Based on barnase fused with a MYC epitope, immunotoxins able to selectively downregulate MYC-specific B cells were developed. They were shown to have an important influence on the development of both systemic and organ-specific autoimmune diseases (Stepanov et al., 2011; **Table 4**).

The Makarov and Ilinskaya groups studied the cytotoxicity of binase using a number of cell lines, distinguished by expressed oncogenes: myeloid precursors FDC-P1; FDC-P1-N822K cells expressing the KIT oncogene; transduced FDC-P1 cells expressing the AML1-ETO oncogene; transduced FDC-P1-N822K cells expressing the AML1-ETO and KIT oncogenes; cells of acute myeloid leukemia Kasumi-1, also expressing both oncogenes, and human ovarian cancer cells (Ilinskaya et al., 2007; Mitkevich et al., 2011; Garipov et al., 2014; **Table 4**). The sensitivity of cells to binase was shown to be dependent on the level of oncogenes, and that the Kasumi-1 cell line was the most sensitive (Mitkevich et al., 2011). It was found that the high expression level of the oncogene KIT also increased the sensitivity of tumour cells to binase (Mitkevich et al., 2010b), suggesting that oncogenic mRNAs may be targets for binase. However, binase exhibits antitumor activity not only due to its own ribonuclease activity, but also due to its ability to bind to certain proteins. Direct interaction of binase and the oncogenic protein KRAS was demonstrated and resulted in the stabilisation of the inactive KRAS conformation and inhibition of MAPK signalling (Ilinskaya et al., 2016).

The Makarov and Zenkova groups first demonstrated the ability of binase to retard primary tumour growth and inhibit metastases formation in experimental murine tumour models: Lewis lung carcinoma, drug resistant lymphosarcoma RLS_40_, and melanoma B16 (Mironova et al., 2013a; **Table 4**). Treatment of animals bearing tumours of various histogenesis with binase leads to significant retardation of primary tumour growth and dissemination. The therapy was also found to have general systemic, immunomodulatory, and hepatoprotective effects and did not induce inflammatory response in the organism (Mironova et al., 2013a; Sen’kova et al., 2014).

## Conclusion and Critical Point of View

Cell transformation, uncontrolled proliferation, increased migration, and invasion are multistage processes, at all steps of which tumour-associated RNAs take part. The correct balance between intracellular RNAs encoding oncogenes and RNA encoding tumour suppressors, as well as the balance between regulatory oncogenic or oncosuppressive miRNAs, determines the normal cell phenotype, and the imbalance of this equilibrium leads to their transformation. Extracellular tumour-associated coding and regulatory RNAs make a significant contribution to tumour progression through distant transfection of normal cells and the formation of new tumour foci. So named “house-keeping” endogenous ribonucleases are involved in the control of RNA homeostasis and the discarding of aberrant RNAs thus providing the proper functioning of RNA orchestra in the cell. Therein, an alteration of the expression or activity of endogenous nucleases leads to their failure in RNA turnover as well as changes in the profile of regulatory RNAs, which leads to oncogenesis and progression of the tumour.

Thus, it is obvious that exogenous RNases, whose cytotoxic activity was discovered at a time when the era of understanding the role of regulatory and coding RNA in oncogenesis was just beginning, are now recognized participants in control of cell transformation and events of tumor progression. Large amount of data is accumulated confirming that tumour-associated RNAs inside the tumour cell and in the pool of circulating exRNAs, whose levels are significantly increased in the process of tumour progression, can be the targets for exogenous RNases. Exogenous nucleases reducing the amount of both intracellular and circulating tumour-associated RNAs may maintain normal untransformed state of the cell and decrease the rate of tumour dissemination. Thus, exogenous RNase do not at all play the role of a “scavenger,” equally likely cleaving all asseccible RNAs, but the role of a supervisor over the tumor development.

In connection with a certain selectivity of natural RNases to RNAs, they can be considered as useful tools for searching for tumor-associated RNA targets. This strategy let to identify a wide range of RNA targets for selective shutdown in a tumor cell. Numerous studies of the antitumor activity of natural RNases, including preclinical ones, in the long term may provide completely new anticancer drugs that work not only at the cell level, but also at the level of organism.

## Author Contributions

NM analyzed published data and prepared the manuscript. VV revised and corrected the manuscript.

## Funding

This work was funded by the Russian State funded budget project of Institute of Chemical Biology and Fumdamental Medicine SB RAS # АААА-А17-117020210024-8 and grant RFBR (no. 17-00-00059 and 17-00-00062).

## Conflict of Interest Statement

The authors declare that the research was conducted in the absence of any commercial or financial relationships that could be construed as a potential conflict of interest.
